# Research Perspectives on the Regulation and Physiological Functions of FGF21 and its Association with NAFLD

**DOI:** 10.3389/fendo.2015.00147

**Published:** 2015-09-23

**Authors:** Takeshi Inagaki

**Affiliations:** ^1^Division of Metabolic Medicine, Research Center for Advanced Science and Technology, The University of Tokyo, Tokyo, Japan

**Keywords:** FGF21, NAFLD, NASH, ER stress, chronic inflammation, epigenetic regulation

## Abstract

Fibroblast growth factor 21 (FGF21) is a metabolic hormone primarily secreted from the liver and functions in multiple tissues. Various transcription factors induce FGF21 expression in the liver, which indicates that FGF21 is a mediator of multiple environmental cues. FGF21 alters metabolism under starvation conditions, protects the body from energy depletion, and extends life span. Pharmacological administration of FGF21 alleviates dyslipidemia and induces weight loss in obese animals. In addition to the well-studied functions of FG21, several lines of recent evidence indicate a possible link between FGF21 and non-alcoholic fatty liver disease (NAFLD). High serum levels of FGF21 are associated with NAFLD and its risk factors, such as endoplasmic reticulum stress and chronic inflammation. In addition, FGF21 alleviates the major risk factors of NAFLD, including obesity, dyslipidemia, and insulin insensitivity. Thus, FGF21 is a potential drug candidate for diseases, such as NAFLD, dyslipidemia, and type 2 diabetes. In this review, the research perspectives of FGF21 and therapeutic potencies of FGF21 as a modulator of NAFLD are summarized.

## Endocrine FGFs

Fibroblast growth factors (FGFs) constitute a large family of signaling proteins. In vertebrates, 22 members of the FGF family (FGF1-23; mouse FGF15 is an ortholog of human FGF19) have been identified (1, 2). Most members of this family mainly work as autocrine or paracrine factors by activating single-pass membrane-spanning FGF receptors (FGFR1–4) on the cell surface in the presence of heparan sulfate (3, 4). FGF15/19, FGF21, and FGF23 work as hormones by binding to the FGFR, which requires either β-Klotho for binding of FGF15/19 and FGF21 or Klotho for FGF23 binding. This distinct mechanism by which the endocrine FGFs alter the binding affinity to the FGFR and the specific isoforms of FGFRs would explain the tissue specificity of endocrine target tissues and cell types.

## Expression and Secretion of FGF21

Although FGF21 is mainly expressed in the liver, it is also found in the pancreas, white adipose tissue (WAT), and stressed muscle tissues. Besides being an autocrine/paracrine factor, FGF21 is also secreted into the bloodstream and acts as a hormone. A recent study revealed that most, if not all, of the circulating FGF21 is derived from the liver (5). Markan et al. showed that plasma FGF21 level was completely abolished in liver-specific FGF21 knockout mice, whereas adipose-specific FGF21 knockout mice showed normal levels of plasma FGF21. FGF21 functions physiologically and pharmacologically to maintain energy homeostasis. It improves insulin sensitivity and glycolipid metabolism and reduces hepatic lipid accumulation.

**Figure 1:**
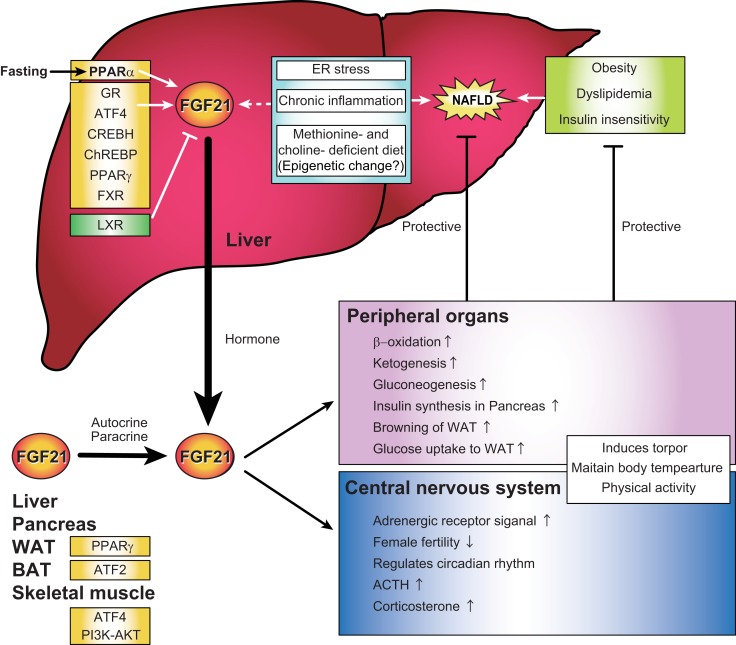
**Regulations and functions of FGF21 signal representing a possible link between FGF21, non-alcoholic fatty liver disease, endoplasmic reticulum stress, and chronic inflammation in the liver**. FGF21 is expressed in the liver, pancreas, adipose tissue, and muscle in response to various environmental cues. Several recent evidences indicate that hepatic FGF21 expression is also regulated by endoplasmic reticulum stress, chronic inflammation, and epigenetics, all of which are correlated with the pathogenesis of non-alcoholic fatty liver disease (NAFLD). Plasma FGF21 is primarily secreted from the liver and acts in both peripheral organs and the central nervous system as a regulator of multiple biological contexts that improve NAFLD, obesity, dyslipidemia, and insulin insensitivity.

Fibroblast growth factor 21 gene transcription in the liver is regulated by nuclear receptor peroxisome proliferator-activated receptor (PPAR) α, which plays critical roles in fasting response (Figure 1) (6–8). Under the regulation of PPARα, hepatic FGF21 mRNA is highly expressed (~20-fold) in fasting mice. Other reports have shown that the hepatic expression of FGF21 is also regulated positively or negatively by glucocorticoid receptor (GR) (9), activating transcription factor 4 (ATF4) (10–13), cAMP-responsive element-binding protein H (CREBH) (14), carbohydrate response element-binding protein (ChREBP) (15), PPARγ (16, 17), farnesoid X receptor (FXR) (18), and liver X receptor (LXR) (19, 20) under various conditions (Figure 1) [reviewed in Ref. (21)]. In skeletal muscle, the expression of FGF21 was reported to be regulated by ATF4 under the conditions of mitochondrial dysfunction (12) and by PI3K–AKT signaling pathway (22). In brown adipose tissue (BAT), FGF21 is regulated by ATF2 (23), while in WAT, it is regulated by PPARγ (24). These known regulatory pathways indicate diverse FGF21 functions under the conditions, such as depletion of energy sources, ER stress, mitochondrial dysfunction, and on exposure to cold environment. This review will not only focus on the well-studied functions of FGF21 in glucose and lipid metabolism but also discuss current reports that suggest a possible link between FGF21 and fatty liver disease, ER stress or chronic inflammation to present research perspectives into the novel FGF21 functions in this broad and developing area of research.

## FGF21 in Energy Homeostasis

During the course of starvation, there is a gradual transition of the energy source of our body from glucose and glycogen to fat, protein, and acetate (25, 26). Glucose is an important energy source for the body, especially for the brain where β-oxidation of fatty acids is unavailable. During the first stage of fasting (several hours), glucose is supplied by glycogenolysis. Subsequently, gluconeogenesis occurs to maintain the level of glucose through various pathways, such as the pathway in which phosphoenolpyruvate carboxykinase (PEPCK) catalyzes the conversion of oxaloacetate to phosphoenolpyruvate, and the pathway in which glycerol kinase and glycerol 3-phosphate dehydrogenase catalyze the conversion of glycerol, a product of adipose tissue lipolysis, to dihydroxyacetone phosphate. Under prolonged fasting, triglyceride is metabolized into glycerol and free fatty acids through β-oxidation. The free fatty acids are converted to ATP and acetyl-coA, which are metabolized to ketone bodies, such as acetone, acetoacetate, and β-hydroxybutyrate. Ketone bodies are used as an energy source under severe starvation because long-chain fatty acids cannot pass through the blood–brain barrier. FGF21 reduces glycogenolysis and induces β-oxidation, ketogenesis, and gluconeogenesis even under normal feeding conditions (6, 27). It is an important rheostat that shifts the energy source from glycogen to fat and ketone bodies for survival under energy deprivation status.

Studies using mice model have revealed that FGF21 induces lipolysis and maintains plasma glucose level by regulating expressions of hormone-sensitive lipase (HSL) and adipose triglyceride lipase (ATGL) in WAT and ectopic expressions of pancreatic lipases in the liver (6). The production of glucose through gluconeogenic enzymes, such as PEPCK and glucose-6-phosphatase (G6Pase), is partially mediated by the transcriptional co-activator PPAR-γ co-activator-1 α (PGC1-α), whose expression is regulated by FGF21 (27). Administration of FGF21 to the obese rodent model was shown to suppress glucose, insulin, and triglycerides levels in the plasma and triglycerides levels in the liver, and enhance lipid usage. It has been reported that FGF21 induces glucose transfer into the adipocytes by increasing GLUT1 expression (28), increases the quantity of pancreatic islets and insulin secretion from each pancreatic islet (29), and suppresses plasma glucagon levels (28). FGF21 also exerts anti-obesity effects through regulation of energy expenditure in BAT by stimulating sympathetic nerve activity through a mechanism that depends on the neuropeptide corticotrophin-releasing factor (CRF) (30).

During prolonged starvation, the body adapts by preserving critical survival systems, such as heartbeat and brain activity, while reducing other energy expenditures, including growth, reproduction, and maintenance of skeletal stature. FGF21 inhibits growth hormone (GH) signaling by inhibiting the Janus kinase/signal transducer and activator of transcription (Jak-STAT) signaling pathway (31). This results in suppression of insulin-like growth factor gene (Igf-1) and other GH target genes in the liver. FGF21 also inhibits female fertility by suppressing the vasopressin–kisspeptin signaling cascade at the superchiasmatic nucleus (SCN) in the hypothalamus, which leads to the inhibition of the proestrus surge of luteinizing hormone (32). In addition, FGF21 promotes bone loss (33, 34). This is consistent with a previous report that FGF21 induces the stimulation of the sympathetic nervous system, which is known to induce bone loss (30). FGF21 induces a hibernation-like state of reduced body temperature in rodents called torpor. This is induced by fasting, accompanied by increased ketogenesis, and associated with induction of pancreatic lipases in the liver (6, 35). FGF21 transgenic mice show all of the above characteristics. Furthermore, transgenic overexpression of FGF21 in mice extends their lifespan without having to reduce food intake or affecting the nicotinamide adenine dinucleotide (NAD^+^) metabolism or AMP-activated protein kinase (AMPK) and mammalian target of rapamycin (mTOR) signaling (36). It has also been reported that FGF21 works on the SCN and dorsal vagal complex of the hindbrain to regulate circadian rhythm, which is important for the adaptive starvation response (37). FGF21 has also been reported to be induced in ground squirrels during hibernation, although the administration of FGF21 did not induce hibernation in them (38). These facts indicate that FGF21 plays important roles in survival and longevity by maintaining energy homeostasis.

Besides the induction of torpor by FGF21 under fasting conditions to maintain the energy source, recent reports claim a link between FGF21 and thermogenesis. Thermogenic stimulations induce mRNA expression and secretion of FGF21 from BAT (23, 39–42). FGF21 is reported to stimulate browning of the inguinal WAT in addition to increasing *Ucp1* expression in both BAT and WAT (39, 43–45). The correlation of FGF21 with browning has been suggested as the mechanism by which FGF21 improves metabolic disorders, such as obesity and type 2 diabetes. However, two recent studies using *Ucp1*-null mice treated with a long-acting FGF21 analog claim that FGF21 does not require either UCP1 or browning of WAT to improve body weight and glucose homeostasis (46, 47). Further investigations would reveal more details of the role of FGF21 in heat production.

## Link between FGF21 and NAFLD

Non-alcoholic fatty liver disease (NAFLD) is one of the most common forms of chronic liver diseases; understanding its epidemiology and further improvement of its diagnostic evaluation and treatment is important. NAFLD ranges from hepatic steatosis (fatty liver) to non-alcoholic steatohepatitis (NASH) and liver cirrhosis (48). Currently, the pathogenesis of NAFLD is attributed to a multi-hit process that involves lipotoxicity, oxidative stress, ER stress, a chronic inflammatory state, and mitochondrial dysfunction. The major risk factors for NAFLD are obesity, dyslipidemia, and insulin insensitivity, which have been shown to be improved by FGF21. FGF21 reverses hepatic steatosis, counteracts obesity, and improves insulin insensitivity (48). Methionine- and choline-deficient diet (MCD) is a model for NASH in rodents (49). It is also known that methionine-deficient diet enhances lipolysis in WAT, and decreases glucose (49, 50). MCD as well as FGF21 increase ATGL and HSL activities in all adipose depots (51). In addition, high serum levels of FGF21 are associated with hepatic steatosis (52–56). These facts indicate that FGF21 level is regulated under the NAFLD condition and may be involved in the protection from NAFLD progression by reversing the steatosis and improving the metabolic energy status (Figure 1). FGF21 has preventive function against lipotoxicity, oxidative stress, ER stress, and chronic inflammatory state, although the mechanisms underlying its functions are mostly unknown.

## Epigenetic Regulation of FGF21 during Development of NAFLD

Although the increased hepatic FGF21 expression is thought to be responsible for the elevated serum levels of FGF21 in patients with NAFLD (10), the underlying molecular mechanism is unclear. Besides the FGF21 induction seen in the MCD-induced NASH model, the methionine-deficient only diet also increases FGF21 in the serum of rodents (49, 50). This interesting finding suggests that a methionine-dependent epigenetic regulation of FGF21 transcription might be involved in the pathogenesis and prevention of NAFLD (Figure 1). It is known that intracellular metabolites, including *S*-adenosyl-methionine (SAM), a-KG, flavin adenosine dinucleotide (FAD), acetyl-CoA, and NAD^+^, are required as substrates for epigenetic modifying enzymes (57, 58). SAM is a methyl donor required for the maintenance of DNA methylation and histone methylation.

## ER Stress and FGF21

The endoplasmic reticulum (ER) plays a crucial role in the folding of proteins and only properly folded proteins are transported to the Golgi apparatus. ER stress is induced by the accumulation of unfolded and/or misfolded proteins in the ER lumen and causes the unfolded protein response (UPR) of the cell. The UPR restores ER homeostasis and function by halting protein translation, degrading misfolded proteins, and activating signaling pathways to increase chaperones for protein folding. ER stress plays a critical role in metabolic homeostasis, as shown in a number of diseases, including NAFLD, obesity, and type 2 diabetes. Recent studies have shown that both triglycerides- and tunicamycin-induced ER stress stimulates FGF21 expression in hepatocytes and serum levels of FGF21 (10, 59–62). Among the three major transducers of the UPR, namely PERK, IRE1, and ATF6, FGF21 is reported to be regulated by PERK and IRE1 (10). Activation of PERK inactivates the translation initiation factor eIF2α by phosphorylation at Ser51. This inactivation paradoxically induces translation of the transcription factor ATF4, which directly regulates FGF21 expression through two binding sites in the FGF21 promoter region. In addition to transcriptional regulation by ATF4, FGF21 is also directly regulated by proapoptotic protein CCAAT enhancer binding protein homologous protein (CHOP) and XBP1 (59, 61). Activation of IRE1, which is a transducer of UPR, generates an active form of transcription factor XBP1 by inducing site-specific splicing and in turn induces FGF21 expression (61). In addition, transcription of β-Klotho coding gene (klb) is associated with increased ER stress in diet-induced obese patients and rodents, and ATF4 signaling pathway is essential for induction of the gene expression of klb, which is mediated by ER stress (63). These findings suggest that FGF21 is a possible link between increased cellular stress and NAFLD (10). However, the detailed mechanism underlying the FGF21 function in NAFLD still needs to be elucidated.

## FGF21 and Its Role in Chronic and Acute Inflammation

The pathogenesis of NAFLD is partially attributed to a chronic inflammatory state. FGF21 may be associated with the chronic inflammation in NAFLD. PPARα, the potent FGF21 regulator in the liver, regulates the expression of genes involved in chronic inflammation as well as fatty acid metabolism in the liver. In the heart, an elective cardiac surgery that induces systemic inflammatory response is accompanied by a marked increase in circulating FGF21, TNF-α, and insulin levels (64). Serum FGF21 levels are also higher in seropositive rheumatoid arthritis (RA) compared with seronegative RA (65). In type 2 diabetic rat models, FGF21 administration ameliorates inflammation biomarkers (66). In terms of FGF21 signaling, inflammatory cytokine TNF-α impairs FGF21 signal by suppressing the expression of β-Klotho in adipocytes (67). These findings point to a potential link between FGF21 and chronic inflammation in the microvasculature. In the acute phase response (APR), FGF21 is reported to modulate the levels of ketone bodies and free fatty acids in response to lipopolysaccharide (LPS) and is protective against the toxic effect of LPS and sepsis. APR inducers, such as LPS, zymosan, and turpentine, increase FGF21 expression in the adipose tissue, muscle, and serum, while they suppress FGF21 expression in the liver (68). These observations suggest that FGF21 is closely involved in the inflammatory response, although these expression regulations of FGF21 by APR are not observed in the cell culture models. It is possible that LPS-induced liver injury may affect both synthesis and degradation of FGF21 in the liver.

## Concluding Remarks

Fibroblast growth factor 21 is an important regulator of metabolism and is a potential therapeutic drug candidate. In addition to its therapeutic benefits, FGF21 could be a prognostic indicator and/or diagnostic marker of metabolic imbalance and other homeostatic disorders. FGF21 is reported to be involved in various pathological conditions, including fatty liver disease, ER stress, and chronic inflammation, as discussed in this manuscript. Despite the multitude of effects of FGF21, whether administration or induction of FGF21 or activation of FGFRs could be pharmacologically beneficial is still controversial. While metabolic profiles are drastically improved by FGF21 in obese rodent models, the results considerably differ among species, thus questioning the appropriateness of FGF21 for the treatment of human diseases, such as obesity, hyperlipidemia, and type 2 diabetes (2). Gaich et al. studied the effects of an FGF21 analog in a randomized, placebo-controlled, double-blind, proof-of-concept trial in obese human patients with type 2 diabetes (69). They showed that a 28-day treatment with the FGF21 analog LY2405319 (LY) improved the plasma levels of low-density lipoprotein cholesterol, triglycerides, high-density lipoprotein cholesterol, and fasting insulin in these patients.

It is necessary to establish the appropriate concentration range of FGF21 for its therapeutic administration, since serum FGF21 concentrations show wide individual variation (2). Galman et al. reported that the fasting serum FGF21 levels checked in the morning in 76 healthy subjects are varied 250-fold from 21 to 5300 pg/ml (70). The serum FGF21 levels in patients with NAFLD or type 2 diabetes were also within the range (52, 71). The diurnal variation of FGF21 is still controversial (2), while a previous study has reported that FGF21 concentration in humans peaks in the early morning (72). Gaich et al. reported that the mean concentration of LY, which is indistinguishable from native FGF21, increased in a dose-dependent manner and was 10- to over 100-fold greater than that observed in the previous reports mentioned above (2, 69, 71).

Because FGF21 has various physiological and pharmacological functions, the large dose of FGF21 required for its *in vivo* functions could cause unexpected side effects. There are several concerns with regard to the side effects of FGF21 [reviewed in Ref. (73, 74)]. FGF21 inhibits GH–IGF1 axis in the liver (31) and antagonize the effects of GH in chondrocytes (75), raising the possibility that FGF21 blocks somatic growth. It also promotes bone loss in mice models indicating a risk of inducing osteoporosis (33). As previously mentioned in this manuscript, FGF21 also inhibits female fertility (32). Therefore, further studies to elucidate the physiological and pharmacological roles of FGF21 in detail are warranted so as to provide important insights for the use of FGF21 as therapeutic drugs to treat metabolic disorders.

## Conflict of Interest Statement

The author declares that the research was conducted in the absence of any commercial or financial relationships that could be construed as a potential conflict of interest.
